# Predictors of institutional delivery service utilization among women of reproductive age in Senegal: a population-based study

**DOI:** 10.1186/s13690-020-00520-0

**Published:** 2021-01-11

**Authors:** Betregiorgis Zegeye, Bright Opoku Ahinkorah, Dina Idriss-Wheelr, Olanrewaju Oladimeji, Comfort Z. Olorunsaiye, Sanni Yaya

**Affiliations:** 1 HaSET Maternal and Child Health Research Program, Shewarobit Field Office, Shewarobit, Ethiopia; 2 The Australian Centre for Public and Population Health Research (ACPPHR), Faculty of Health, University of Technology Sydney, Sydney, Australia; 3 Interdisciplinary School of Health Sciences, University of Ottawa, Ottawa, Ontario Canada; 4 Department of Public Health, Walter Sisulu University, Mthatha, Eastern Cape South Africa; 5 Faculty of Health Sciences, Durban University of Technology, Durban, South Africa; 6 Department of Family Medicine and Public Health, Faculty of Medicine, University of Botswana, Gaborone, Botswana; 7 Department of Public Health, Arcadia University, Glenside, PA USA; 8 Faculty of Social Sciences, School of International Development and Global Studies, University of Ottawa, 120 University Private, Ottawa, ON K1N 6N5 Canada; 9 The George Institute for Global Health, Imperial College London, London, UK

**Keywords:** Institutional delivery, Women, Reproductive age, Senegal, Maternal health, Global health, Sub-Saharan Africa

## Abstract

**Background:**

In Senegal, sub-Saharan Africa, many women continue to die from pregnancy and childbirth complications. Even though health facility delivery is a key intervention to reducing maternal death, utilization is low. There is a dearth of evidence on determinants of health facility delivery in Senegal. Therefore, this study investigated the predictors of health facility-based delivery utilization in Senegal.

**Methods:**

Data from the 2017 Senegal Continuous Survey were extracted for this study, and approximately 11,487 ever-married women aged 15–49 years participated. Chi-square test was used to select significant variables and multivariable logistic regression analysis was performed to identify statistically significant predictors at a 95% confidence interval with a 0.05 *p*-value using Stata version 14 software.

**Results:**

Facility-based delivery utilization was 77.7% and the main predictors were maternal educational status (primary school Adjusted Odds Ratio [aOR] = 1.44, 95% CI; 1.14–1.83; secondary school aOR = 1.62, 95% CI; 1.17–2.25), husband’s educational status (primary school aOR = 1.65, 95% CI; 1.24–2.20, secondary school aOR = 2.17, 95% CI; 1.52–3.10), maternal occupation (agricultural-self-employed aOR = 0.77, 95% CI; 0.62–0.96), ethnicity (Poular aOR = 0.74, 95% CI; 0.56–0.97), place of residence (rural aOR = 0.57, 95% CI; 0.43, 0.74), media exposure (yes aOR = 1.26, 95% CI; 1.02–1.57), economic status (richest aOR = 5.27, 95% CI; 2.85–9.73), parity (seven and above aOR =0.46, 95% CI; 0.34–0.62), wife beating attitude (refuse aOR =1.23, 95% CI; 1.05–1.44) and skilled antenatal care (ANC) (yes aOR = 4.34, 95% CI; 3.10–6.08).

**Conclusion:**

Uptake of health facility delivery services was seen among women who were educated, exposed to media, wealthy, against wife-beating, attended ANC by skilled attendants and had educated husbands. On the other hand, women from ethnic groups like Poular, those working in agricultural activities, living in rural setting, and those who had more delivery history were less likely to deliver at a health facility. Therefore, there is the need to empower women by encouraging them to use skilled ANC services in order for them to gain the requisite knowledge they need to enhance their utilization of health facility delivery, whiles at the same time, removing socio-economic barriers to access to health facility delivery that occur from low education, poverty and rural dwelling.

## Background

Although significant progress has been made in the last two decades, worldwide, approximately 295, 000 women died from pregnancy and childbirth in 2017, which is unacceptably high [1]. Eighty-six percent of global maternal deaths occur in sub-Saharan Africa and Southern Asia [2]. However, there is evidence that in most countries where over 80% of deliveries are attended by health professionals, the Maternal Mortality Rate (MMR) is below 200 per 100,000 live births. In 2017, sub-Saharan Africa alone accounted for roughly two-thirds (196,000) of maternal deaths, while Southern Asia accounted for nearly one-fifth (58,000) [3].

Evidence shows that in low resourced countries in Africa, including Senegal, the cause of maternal death is often linked to non-use of health facility during delivery (also called institutional delivery) [4, 5]. Additional factors include poor socioeconomic status, inadequate access to health facility delivery, ineffective referral systems for women in obstetric emergencies, as well as long distance to health facilities among women who dwell in rural areas [6–8].

The Safe Motherhood Initiative considers institutional delivery as a crucial element in its emphasis on ensuring the availability and accessibility of skilled care during pregnancy and childbirth [9]. Utilization of institutional delivery has been considered as one of the most essential interventions to reduce maternal death, with the proportion of women who utilize skilled assistance during delivery regarded as a key indicator in every country’s health plan [10–13]. A woman who gives birth at a health facility can receive sufficient medical care during childbirth, which helps to reduce preventable maternal and neonatal deaths [13–15].

In 2018, 81% of the mothers delivered by skilled birth attendants globally; the percentages were 77% and 60% in Southern Asia and sub-Saharan Africa, respectively [2]. Beyond this, large disparities in uptake of skilled birth attendant services are seen. Globally, women in the richest subgroups are nearly twice as likely to be delivered by skilled birth attendants compared to the poorest women (91% vs 51%). Similarly, in sub-Saharan Africa, women in the richest subgroups are 2.4 times more likely to be delivered by skilled birth attendants than the poorest (88% vs 37%) [2]. In Senegal, where the MMR is still very high (315/100,000 live births in 2017), some improvements have been made to increase access to health facility delivery from 41.4% in 1986 to 68.4% in 2017 [16].

Prior studies have reported barriers to using skilled delivery services that included behavioral, cultural, and economic factors as well as issues of inaccessibility to health facility, insufficient infrastructure and limited skilled human resources for healthcare at the community level [17–22]. Numerous scholars have also described the rationale behind non-utilization of health facility delivery in developing countries [17, 22–26], and few studies have been conducted in Senegal related to facility delivery. However, it focused merely on coverage of health facility delivery [27, 28], one [29] or few [4] factors, restricted to one region [29, 30] and used old data (1997 & 2014) [4] (2011) [29].

Recognition of drivers of utilization of the facility-based delivery is a crucial step in reducing maternal and neonatal deaths. It can contribute to the development of interventions and policy changes for key populations to improve health outcomes for women and children, particularly in the context of high MMR in low-and middle-income countries [31]. The aim of this study was to examine the drivers of health facility-based delivery in Senegal using recent data from the 2017 Senegal Continuous Survey.

## Methods

### Study setting

Senegal, located in West Africa, is well-known as the “Entry to Africa” [32, 33]. Up to half of its 15.4 million people (as of 2016) live in and around Dakar and other urban areas [33]. Since 1960, three very non-violent political changes have taken place, ensuring its stability [34]. The nation’s economic growth, reported at 6% growth rate in 2018, looks promising for the future [34]. According to available data in 2011 by the World Bank, 38% of Senegal’s population lives on less than $1.90 per day [34].

Senegal’s health system is a hierarchical structure, with each of the 14 regional medical offices in charge of the provision and supervision of healthcare within the regions. There are also health districts which usually consist of one health center linked to rural health posts, some of which supervise the allied health huts [35]. There are also community-level facilities known as health huts, usually operated by a community health worker employed by community health committees [36].

### Data source and sampling procedure

We used the most recent (2017) Senegal Continuous Survey (SCS) for this analysis [37]. Sampling for the 2017 SCS was done using a stratified, two-stage cluster sampling design to provide estimates for essential population and health indicators for the country. Large geographic settings known as enumeration areas (EAs) were selected in the first stage through Probability Proportional to Size (PPS). The survey included a total of 8800 (4092 in urban areas and 4708 in rural) households and a total of 16,787 women (15–49 years of age) and 6977 Men (15–59 years of age) were interviewed [37]. Household listing was completed in each EA to ready the sampling frame. Selected participants were questioned using standard and country-specific questionnaire modules covering a wide range of health topics. For this study, we included 11,487 currently married women aged 15–49 years with a birth, for the most recent live births in the 5 years preceding the survey [37] from the kids (children) recode file (KR). The survey is publicly available on the DHS website (www.dhsprogram.com).

### Variables selection

#### Dependent variable

Place of delivery was the outcome variable in this study and was grouped into health facility delivery (deliveries that occurred in a government hospital, government health center/maternity, government health post, mobile government clinic, government field worker, other public sector, private hospital/clinic and other private sectors) and non-health facility delivery (deliveries that occurred at respondents’ or relatives’ homes, or in other places like on the road). Births with missing information were added to the denominator for both the distribution of place of delivery and percentage of all births that occurred in a health facility. The percentage distribution of place of delivery included a separate category for missing values. Despite the fact that data were available for all live births to questioned women in the 5 years preceding the survey, we calculated for only the most recent birth as recommended by DHS guideline.

#### Independent variables

Several individual and community level explanatory variables were incorporated from previous studies [17, 21, 23, 24, 38–43] due to their role in contributing to increase or decrease in the use of facility delivery. The independent variables were maternal age (15–19, 20–24, 25–29, 30–34, 35–39, 40–44, 45–49), maternal educational status (no formal education, primary school, secondary school, higher), maternal occupation (not working, sales and services, agricultural-self-employed, other), husband education (no formal education, primary school, secondary school, higher), husband occupation (not working, professional/technical or managerial, sales and services, agricultural-self-employed, skilled manual, unskilled manual, other), religion (Muslim, Christian), ethnicity (Wolof, Poular, Serer, Mandingue/Soce, Diola, Soninke, other Senegalese, other), region (Dakar, Ziguinchor, Diourbel, Saint-Louis, Tambacounda, Kaolack, Thios, Louga, Fatick, Kolda, Matam, Kaffrine, Kedougou, Sedhiou), and wealth index (poorest, poorer, middle, richer, richest).

We looked at media exposure (if the respondent was exposed to any of the three types; read newspaper, listened to radio or watched television for at least less than once a week it was coded as yes, and otherwise, no), place of residence (urban, rural), and parity (<=2, 3–4, 5–6, 7+). Decision making power was also included; we looked to see if the respondent had no decision-making power, she alone made decisions, or if she made decisions together with her husband. There were three decision making parameters; decision making about her health, to purchase large household items, to visit family/relatives. We coded “no decision making” if only the husband or other family members made decisions; we coded “decision making one” if the respondent had decision making power either alone or together with her husband on two of the above decision-making parameters; and we coded “decision making power two” if the respondent made decisions alone or together with her husband on all three decision making power parameters. Attitude toward wife beating was assessed as “refused” if the respondent disagreed with all five of the wife beating circumstances presented (burning the food while cooking, arguing with husband, going to visit family without husband permission, neglecting children, refusing to have sex with her husband), and “accepted” if she agreed to any of the five wife beating parameters. We included the use of skilled antenatal care (ANC); if the women had ANC follow up by a skilled attendant (i.e. doctor, midwife, nurse) we coded as yes, if not we coded as no.

### Data analysis

The participants’ socio-demographic characteristics were computed. Chi-square test was performed to identify variables that showed significant associations with the outcome variable at *p*-value less than 0.05 cut point. These variables were entered into the multivariable logistic regression model. Results of the multivariable logistic regression were reported using adjusted odds ratios (aORs) at a 95% confidence interval. Data was analyzed using Stata version 14 software (Stata Corp, College Station, Texas, USA). Weighting was applied using the guidelines provided in the user manual (https://www.dhsprogram.com/pubs/pdf/DHSG4/Recode7_DHS_10Sep2018_DHSG4.pdf), while the ‘SVY’ command was used to account for the complex sampling design.

### Ethical clearance

Since we used secondary data from SCS dataset which is available publicly, we did not need further ethical approval to use the data. However, in addition to obtaining the participants consent prior to survey, the ICF international strictly followed the ethical standards collaborating with the concerned country’s Ethical Review Board to ensure the DHS data collection process was in line with the U.S. Department of Health and Human Services regulations for the respect of the right of human subjects.

## Results

In this study, a total of 11,487 married women were included, of whom 2926 (25.5%) were 25–29 years old, and more than three-fifths (67.5%) were rural residents (Table 1). More than two-thirds (67.2%) of the participants and three fourth (75.6%) of their partners had no formal education. Nearly two-fifth (38.9%) of the respondents were unemployed, whereas more than one fourth (28.3%) of them were self-employed in agriculture. The majority (97.7%) of the participants were from the Muslim faith, and about 87.5% of the respondents were from Poular (33.3%), Wolof (30.9%), Serer (13.7%) and Mandingue/Soc (10.1%) ethnicities. Most of the participants (88.9%) had exposure to media, at least once a week to newspaper, radio or television.

**Table 1:** Respondents’ socio-economic characteristics, 2017 Senegal Continuous Survey

Variable	Frequency	Percent	Place of delivery		Chi-square, ***p***-values
Health facility delivery			Not health facility Frequency (%)	Health facility, Frequency (%)	
No	3074	22.33			
Yes	8413	77.67			
**Maternal age**					χ2 = 38.03, *p* < 0.001
15–19	561	4.88	125 (22.28)	436 (77.72)	
20–24	2202	19.17	526 (23.89)	1676 (76.11)	
25–29	2926	25.47	789 (26.97)	2137 (73.03)	
30–34	2733	23.79	733 (26.82)	2000 (73.18)	
35–39	1801	15.68	489 (27.15)	1312 (72.85)	
40–44	973	8.47	311 (31.96)	662 (68.04)	
45–49	291	2.53	101 (34.71)	190 (65.29)	
**Maternal educational status**					χ2 = 586.21, *p* < 0.001
No formal education	7719	67.22	2590 (33.55)	5129 (66.45)	
Primary school	2320	20.20	363 (15.65)	1957 (84.35)	
Secondary school	1290	11.23	118 (9.15)	1172 (90.85)	
Higher	155	1.35	1 (0.65)	154 (99.35)	
**Maternal occupation**					χ2 = 586.21, *p* < 0.001
Not working	4474 s	38.95	2590 (33.55)	5129 (66.45)	
Sales and services	2422	21.08	363 (15.65)	1957 (84.35)	
Agricultural-self employed	3245	28.25	118 (9.15)	1172 (90.85)	
Others	1346	11.72	1 (0.65)	154 (99.35)	
**Husband educational status**					χ2 = 494.70, *p* < 0.001
No formal education	8688	75.63	2771 (31.89)	5917 (68.11)	
Primary school	1366	11.89	192 (14.06)	1174 (85.94)	
Secondary school	1025	8.92	87 (8.49)	938 (91.51)	
Higher	408	3.55	24 (5.88)	384 (94.12)	
**Husband occupation**					χ2 = 656.59, *p* < 0.001
Not working	290	2.52	74 (25.52)	216 (74.48)	
Professional/technical or managerial	1147	9.99	144 (12.55)	1003 (87.45)	
Sales and services	2015	17.54	448 (22.23)	1567 (77.77)	
Agricultural-self employed	3179	27.67	1372 (43.16)	1807 (56.84)	
Skilled manual	1606	13.98	309 (19.24)	1297 (80.76)	
Unskilled manual	1805	15.71	383 (21.22)	1422 (78.78)	
Other	1445	12.58	344 (23.81)	1101 (76.19)	
**Religion**					χ2 = 4.69, *P* = 0.030
Muslim	11,224	97.71	3019 (26.90)	8205 (73.10)	
Christian	263	2.29	55 (20.91)	208 (79.09)	
**Ethnicity**					χ2 = 369.60, *p* < 0.001
Wolof	3556	30.96	695 (19.54)	2861 (80.46)	
Poular	3820	33.25	1291 (33.80)	2529 (66.20)	
Serer	1575	13.71	331 (21.02)	1244 (78.98)	
Mandingue/ soce	1159	10.09	442 (38.14)	717 (61.86)	
Diola	345	3.00	29 (8.41)	316 (91.59)	
Soninke	186	1.62	46 (24.73)	140 (75.27)	
Other Senegalese	522	4.54	124 (23.75)	398 (76.25)	
Other	324	2.82	116 (35.80)	208 (64.20)	
**Region**					χ2 = 908.29, *p* < 0.001
Dakar	677	5.89	30 (4.43)	647 (95.57)	
Ziguinchor	497	4.33	56 (11.27)	441 (88.73)	
Diourbel	988	8.60	206 (20.85)	782 (79.15)	
Saint-Louis	730	6.36	158 (21.64)	572 (78.36)	
Tambacounda	920	8.01	436 (47.39)	484 (52.61)	
Kaolack	719	6.26	151 (21.00)	568 (79.00)	
Thiès	878	7.64	97 (11.05)	781 (88.95)	
Louga	835	7.27	196 (23.47)	639 (76.53)	
Fatick	861	7.50	165 (19.16)	696 (80.84)	
Kolda	864	7.52	297 (34.38)	567 (65.63)	
Matam	886	7.71	261 (29.46)	625 (70.54)	
Kaffrine	1111	9.67	357 (32.13)	754 (67.87)	
Kedougou	658	5.73	333 (50.61)	325 (49.39)	
Sedhiou	863	7.51	331 (38.35)	532 (61.65)	
**Wealth index**					χ2 = 7.35, *p* < 0.001
Poorest	3646	31.74	1727 (47.37)	1919 (52.63)	
Poorer	2898	25.23	859 (29.64)	2039 (70.36)	
Middle	2483	21.62	361 (14.54)	2122 (85.46)	
Richer	1477	12.86	87 (5.89)	1390 (94.11)	
Richest	983	8.56	40 (4.07)	943 (95.93)	
**Media exposure**					χ2 = 474.47, *p* < 0.001
No	1278	11.13	667 (52.19)	611 (47.81)	
Yes	10,209	88.87	2407 (23.58)	7802 (76.42)	
**Place of residence**					χ2 = 922.16, *p* < 0.001
Urban	3737	32.53	325 (8.70)	3412 (91.30)	
Rural	7750	67.47	2749 (35.47)	5001 (64.53)	
**Parity**					χ2 = 429.70, *p* < 0.001
< =2	3612	31.44	585 (16.20)	3027 (83.80)	
3–4	3520	30.64	917 (26.05)	2603 (73.95)	
5–6	2396	20.86	786 (32.80)	1610 (67.20)	
7+	1959	17.05	786 (40.12)	1173 (59.88)	
**Decision making**					χ2 = 105.23, *p* < 0.001
No decision making	7716	67.17	2292 (29.70)	5424 (70.30)	
Decision making one	2509	21.84	535 (21.32)	1974 (78.68)	
Decision making two	1262	10.99	247 (19.57)	1015 (80.43)	
**Wife beating attitude**					χ2 = 347.04, *p* < 0.001
Accept	7062	61.48	2320 (32.85)	4742 (67.15)	
Refuse	4425	38.52	754 (17.04)	3671 (82.96)	
**Skilled ANC**					χ2 = 350.81, *p* < 0.001
No	304	3.86	211 (69.41)	93 (30.59)	
Yes	7578	96.14	1701 (22.45)	5877 (77.55)	

Regarding women empowerment, about 7716 (67.2%) had no decision-making power about their own health, to purchase household expenses and to visit family/relatives. Only 2509 (21.8%) and 1262 (10.9%) of the participants had decision-making power on two and three of the above decision-making parameters, respectively. More than three-fifth (61.5%) of the participants accepted wife-beating. The majority (96.1%) of them attended ANC.

### Predictors of health facility delivery

The coverage of health facility delivery among married women was 77.7%. Several individual and community level factors were identified as predictors of health facility deliveries. Compared to women who had no formal education, health facility deliveries were higher by 44% (aOR = 1.44, 95% CI; 1.14–1.83) and 62% (aOR = 1.62, 95% CI; 1.17–2.25) among women who had primary and secondary school education respectively (Table 2).

**Table 2:** Predictors of health facility delivery in Senegal: Evidence from the 2017 Senegal Continuous Survey

Variables	aOR (95% CI)
**Maternal age**	
15–19	1.00
20–24	0.81 (0.57–1.15)
25–29	0.73 (0.49–1.08)
30–34	0.95 (0.64–1.42)
35–39	1.11 (0.70–1.74)
40–44	0.86 (0.54–1.38)
45–49	1.01 (0.55–1.86)
**Maternal educational status**	
No formal education	1.00
Primary school	1.44 (1.14–1.83)**
Secondary school	1.62 (1.17–2.25)**
Higher	21.20 (2.70–166.32)**
**Maternal occupation**	
Not working	1.00
Sales and services	0.97 (0.78–1.21)
Agricultural-self employed	0.77 (0.62–0.96)*
Others	0.88 (0.66–1.17)
**Husband educational status**	
No formal education	1.00
Primary school	1.65 (1.24–2.20)**
Secondary school	2.17 (1.52–3.10)***
Higher	1.49 (0.65–3.43)
**Husband occupation**	
Not working	1.00
Professional/technical or managerial	0.71 (0.41–1.24)
Sales and services	0.96 (0.59–1.57)
Agricultural-self employed	0.92 (0.57–1.47)
Skilled manual	1.28 (0.76–2.14)
Unskilled manual	1.06 (0.66–1.73)
Other	0.98 (0.61–1.56)
**Religion**	
Muslim	1.00
Christian	1.24 (0.66–2.34)
**Ethnicity**	
Wolof	1.00
Poular	0.74 (0.56–0.97)*
Serer	0.96 (0.68–1.36)
Mandingue/ soce	0.86 (0.57–1.27)
Diola	0.93 (0.47–1.84)
Soninke	0.90 (0.42–1.92)
Other senegalese	0.82 (0.50–1.32)
Other	0.59 (0.37–0.93)*
**Region**	
Dakar	1.00
Ziguinchor	1.24 (0.51–2.99)
Diourbel	0.91 (0.46–1.78)
Saint-Louis	1.01 (0.48–2.10)
Tambacounda	0.67 (0.32–1.40)
Kaolack	1.10 (0.48–2.55)
Thies	1.29 (0.66–2.53)
Louga	0.85 (0.41–1.77)
Fatick	1.40 (0.71–2.75)
Kolda	1.26 (0.58–2.70)
Matam	0.97 (0.43–2.16)
Kaffrine	1.51 (0.71–3.22)
Kedougou	0.45 (0.19–1.06)
Sedhiou	0.71 (0.33–1.50)
**Place of residence**	
Urban	1.00
Rural	0.57 (0.43–0.74)***
**Media exposure**	
No	1.00
Yes	1.26 (1.02–1.57)*
**Wealth index**	
Poorest	1.00
Poorer	1.98 (1.61–2.43)***
Middle	2.92 (2.12–4.03)***
Richer	3.88 (2.54–5.91)***
Richest	5.27 (2.85–9.73)***
**Parity**	
0–2	1.00
3–4	0.66 (0.54–0.79)***
5–6	0.53 (0.42–0.67)***
7+	0.46 (0.34–0.62)***
**Decision making**	
No decision making	1.00
Decision making one	1.07 (0.86–1.33)
Decision making two	0.98 (0.73–1.30)
**Wife beating attitude**	
Accept	1.00
Refuse	1.23 (1.05–1.44)**
**Skilled ANC**	
No	1.00
Yes	4.34 (3.10–6.08)***

Notes: *** *P* < 0.001, ** *P* < 0.01, * *P* < 0.05, *ref.* reference

Similarly, health facility delivery among women whose husbands had primary education were approximately 65% (aOR = 1.65, 95% CI; 1.24–2.20) and secondary education were approximately 2.2 times (aOR = 2.17, 95% CI; 1.52–3.10) higher compared to women whose husbands had no formal education. Maternal occupation also had a significant association with health facility delivery with women who were self-employed in agriculture 23% (aOR = 0.77, 95% CI; 0.62–0.96) less likely to deliver in health facilities compared to women who were not working.

In our study, health facility delivery among women from Poular ethnic groups was lower by 26% (aOR = 0.74, 95% CI; 0.56–0.97) as compared to women from Wolof ethnic groups. With place of residence, health facility delivery among women living in a rural settings was lower by 43% (aOR = 0.57, 95% CI; 0.43, 0.74) as compared to urban resident women. Women who had exposure to newspaper, radio or television for at least less than once a week were 26% (aOR = 1.26, 95% CI; 1.02–1.57) more likely than women with no media exposure to delivery in health facilities. Health facility delivery among women in the richer and richest households were 3.8 times (aOR = 3.88, 95% CI; 2.54–5.91) and 5.2 times (aOR = 5.27, 95% CI; 2.85–9.73) higher than for women in the poorest households, respectively.

Compared to women who delivered two or less children, health facility delivery among women who delivered 3–4 and 5–6 or more children, was lower by 34% (aOR = 0.66, 95% CI; 0.54–0.79) and 47% (aOR = 0.53, 95% CI; 0.42–0.67), respectively. Similarly, health facility delivery among women who delivered seven or more children was lower by 54% (aOR = 0.46, 95% CI; 0.34–0.62) as compared to women who delivered two or less children.

Health facility delivery among women who refused wife-beating for any reason was higher by 23% (aOR = 1.23, 95% CI; 1.05–1.44) as compared to women who accepted wife-beating for any reason. Compared to married women who had not received skilled ANC follow-up, health facility delivery among married women who had skilled ANC follow-up was approximately 4.3 times (aOR = 4.34, 95% CI; 3.10–6.08) higher.

## Discussion

Senegal has a high MMR and the utilization of institutional deliveries is low [16]. In this study, we used the 2017 SCS to assess predictors of health facility delivery among married women in Senegal. Similar to previous studies [38, 44], our findings indicate that the use of a health facility for delivery among educated mothers was better than that of mothers with no education. A possible reason could be that educated mothers are more likely to receive care during pregnancy and delivery than uneducated mothers [39]. In addition, educated mothers are believed to be informed on possible signs of obstetric danger, which allows them to seek prompt medical advice [45]. Again, there is an influence of education on women’s healthcare-seeking behavior as it enhances health awareness of services and self-efficacy [38, 44]. Being married to educated husbands also positively predicted the use of health facility-based delivery in our study; previous studies suggest that educated husbands encourage wives to deliver in a health facility [23, 24, 40, 41]. Findings on the association between level of education and the use of health facility delivery indicates the need for government to provide access to education in the country, especially for the girl-child. Apart from the mainstream education in the classroom, media channels such as radio and television can be used as sources of information on the importance of health facility delivery for pregnant women.

We found an association between household wealth status and use of facility delivery and this suggests a dose-response gradient. The odds of facility-based delivery increased as household wealth quintile increased. Findings from our study were similar with previous studies [38]. Reducing poverty or increasing household income generation may lead to improved access to facility-based delivery services [38]. Also, poor households may not be able to pay for cost of transportation in case of referral as distance to the health facility can also be a geographic barrier (i.e. the health facility is far from home [38]. Findings on the disparities in the use of health facility delivery, with poor women less likely to use health facility delivery compared to rich women, show a socio-economic gap that can be bridged by ensuring access to health facility delivery for all, irrespective of wealth status. This can be achieved by removing the financial barriers in access to health facility delivery in the country.

There was a positive association between use of skilled ANC and health facility delivery. This suggests that women who deliver in health facilities are more likely to have adequate knowledge about the risks associated with home delivery and consequences of complicated pregnancy, and understand the essence of delivering at health facilities [46, 47]. It is possible that improving the coverage of skilled ANC services may increase the use of facility delivery [17]. Evidence shows that women who have skilled ANC attendance are more conscious of the risks related to childbirth and therefore have high propensity to deliver at healthcare institutions [48]. Similar findings were seen in Tanzania [42], Ghana [43], Nigeria [49] and Ethiopia [50, 51]. The birth preparedness plan is a key part of the first four ANC visits, a time during which pregnant mothers may be convinced to deliver at health institutions [51]. Study findings call for the need to empower women by encouraging them to use skilled ANC services for them to gain the requisite knowledge to enhance their utilization of health facility delivery.

Consistent with previous work [17, 51], this study confirmed that living in urban settings has a positive influence on institutional delivery. In low-and middle-income countries, mass media communication plays an essential role in enhancing knowledge and awareness about health issues among the general population leading to the promotion of the use of maternity services [17]. Specifically, women who are exposed to media might have a chance to become informed about institutional delivery services and the potential negative consequences of home delivery [51]. Hence, enhancing media exposure will help provide quality healthcare communication that can lead to an improvement in the uptake of health facility deliver services, as supported in a previous study [17].

In line with previous studies [25, 26, 51, 52], our study also showed lower utilization of facility delivery among women who live in rural settings. Women who live in urban settings often have more access to resources and better quality of health services [17, 22]. As well, mothers who live close to health care institutions may be more likely to utilize ANC services, where they will have better access to health education, coupled with ease of transportation [51] and increased awareness of obstetric danger signs that are life-threating for mother and fetus [45]. Findings on the disparities in the use of health facility delivery in rural and urban areas show that the geographical gap can be bridged by ensuring access to health facility delivery for rural dwellers.

This study showed that women working in an agricultural setting are disadvantaged when it comes to delivery at health facilities compared with non-working women, as reported in a prior study [17]. This has been attributed to the monetary and livelihood suffering that occur in the lives of women engaged in agriculture [17]. Another possible reason could be that such women may prioritize and give particular focus to farming communities compared to utilization of maternal healthcare promotion programs.

We found disparities in utilization of facility delivery across ethnic groups with women of the Poular ethnic groups less likely to use health facility delivery services compared to those of the Wolof ethnic group. Possible explanations for the disparities across ethnic groups could be the health insurance use, attitudes of physicians towards minority people, language barriers, transportation cost, low level of education, literacy, poverty and low socio-economic status, familiarity with the health care delivery system, the degree and kind of family support as well as belonging to a lower group in the ethnic hierarchy [53–57].

Consistent with a previous study in Tanzania [58], our study further suggests a strong association between parity and health facility delivery; a mother with more delivery history was less likely to use facility delivery. One possible reason could be that mothers with previous positive birth outcomes from home delivery may choose to deliver again in the home. The other explanation could be, if they had experienced poor quality care, including disrespect by health professionals in a previous facility delivery, they may not want to have another facility delivery.

Our study found decreased likelihood of facility delivery utilization among women who accepted wife-beating as a usual or healthy part of living. Evidence shows women who experience beating are less likely to use health services such as ANC [59], which, may decrease their facility delivery utilization. A possible explanation could be that women may experience beating by the husband when they go to a facility without his permission [60].

A key strength of this study was the use of recent nationally representative data that allows investigation of current barriers for institutional delivery, and in turn, help propose timely interventions to work towards achievement of the Sustainable Development Goal (SDGs). Nevertheless, there are several limitations that should be acknowledged. First, the use of cross-sectional data implies that the authors cannot indicate causality but rather associations with the findings. Again, there is also the likelihood of recall and reporting bias since the data were self-reported. The factors that predict facility delivery are varied and multidimensional, but the choice of the independent variables was limited to those available in the dataset.

## Conclusion

Using the recent 2017 SCS, both the coverage of facility delivery and its individual and community level predictors were comprehensively assessed. Better uptake of facility delivery service was seen among women who were educated, exposed to media, richer or richest, against wife-beating, attended ANC by skilled attendants and had educated husbands. On the other hand, women from some ethnic groups like Poular, those working in agricultural activities, living in rural settings, and those with more history of delivery were less likely to have health facility delivery. Therefore, there is the need to empower women by encouraging them to use skilled ANC services in order for them to gain the requisite knowledge to enhance their utilization of health facility delivery, while at the same time, removing socio-economic barriers to access to health facility delivery that occur from low education, poverty and rural dwelling.

## Data Availability

The datasets generated and/or analyzed during the current study are available in http://dhsprogram.com/data/available-datasets.cfm.

## Abbreviations

AOR: Adjusted Odd Ratio
DHS: Demographic and Health Survey
EA: Enumeration Area
ICF: Inner City Fund
IR: Individual Recode
IRB: Institutional Review Board
PPS: Probability Proportional to Size
SCS: Senegal Continuous Survey
SDG: Sustainable Development Goal
WHO: World Health Organization
